# Integrated agronomic, physiological, microstructure, and whole-transcriptome analyses reveal the role of biomass accumulation and quality formation during Se biofortification in alfalfa

**DOI:** 10.3389/fpls.2023.1198847

**Published:** 2023-07-20

**Authors:** Qingdong Wang, Jinke Hu, Tongbo Lou, Yan Li, Yuhua Shi, Huafeng Hu

**Affiliations:** ^1^ School of Life Sciences, Zhengzhou University, Zhengzhou, Henan, China; ^2^ Henan University of Animal Husbandry and Economy, Zhengzhou, Henan, China; ^3^ Henan Key Laboratory of Bioactive Macromolecules, Zhengzhou, Henan, China; ^4^ Henan Grass and Animal Engineering Technology Research Center, Zhengzhou, Henan, China

**Keywords:** selenium, biofortification, whole-transcriptome RNA-seq, non-coding RNA, miRNA, ceRNA, *Medicago sativa* L

## Abstract

Se-biofortified agricultural products receive considerable interest due to the worldwide severity of selenium (Se) deficiency. Alfalfa (*Medicago sativa* L.), the king of forage, has a large biomass, a high protein content, and a high level of adaptability, making it a good resource for Se biofortification. Analyses of agronomic, quality, physiological, and microstructure results indicated the mechanism of biomass increase and quality development in alfalfa during Se treatment. Se treatment effectively increased Se content, biomass accumulation, and protein levels in alfalfa. The enhancement of antioxidant capacity contributes to the maintenance of low levels of reactive oxygen species (ROS), which, in turn, serves to increase alfalfa’s stress resistance and the stability of its intracellular environment. An increase in the rate of photosynthesis contributes to the accumulation of biomass in alfalfa. To conduct a more comprehensive investigation of the regulatory networks induced by Se treatment, the transcriptome sequencing of non-coding RNA (ncRNA) was employed to compare 100 mg/kg Se treatment and control groups. The analysis identified 1,414, 62, and 5 genes as DE-long non-coding RNAs (DE-lncRNA), DE-microRNAs (DE-miRNA), and DE-circular RNA (DE-circRNA), respectively. The function of miRNA-related regulatory networks during Se biofortification in alfalfa was investigated. Subsequent enrichment analysis revealed significant involvement of transcription factors, DNA replication and repair mechanisms, photosynthesis, carbohydrate metabolism, and protein processing. The antioxidant capacity and protein accumulation of alfalfa were regulated by the modulation of signal transduction, the glyoxalase pathway, proteostasis, and circRNA/lncRNA-related regulatory networks. The findings offer new perspectives on the regulatory mechanisms of Se in plant growth, biomass accumulation, and stress responses, and propose potential strategies for enhancing its utilization in the agricultural sector.

## 1 Introduction

Selenium (Se) is essential for the production of thyroid hormones, defense mechanisms, and antioxidants in animals and humans. Se deficiency should be recognized as a worldwide problem (Genchi et al., 2023). Se levels in the human diet are significantly influenced by the location and cultivation of crops, the fodder fed to animals, and the foods consumed. Because of the negative consequences of inorganic Se, biofortification of crops with natural organic Se by anthropogenic supplementation is a potential technique for increasing human Se intake.

Se is beneficial to the growth of some plants at lower concentrations but detrimental at higher concentrations (Hasanuzzaman et al., 2020). The application of moderate concentrations of selenite and selenate increased both the aboveground and root biomass of wheat (Boldrin et al., 2016). Se fertilization has been observed to enhance the quality of plants. Appropriate concentrations of selenite have been found to enhance both the growth and stress tolerance of plants. The Se content, total polyphenol content, and antioxidant capability of peas were all improved by foliar spraying with Se (Hegedusova et al., 2017). Exogenous Se fertilization increased sunflower photosynthesis while decreasing the negative effects of NaCl stress. Higher-dose Se, on the other hand, wastes resources and pollutes the environment. Furthermore, it frequently causes phytotoxicity and inhibits plant development. Se has been related to harmful effects on crops such as corn (Jiang et al., 2017) and tea (Cao et al., 2018). It is more important to identify the limited range of Se supplementation levels to Se fortification in crop-specific (For example, while and alfalfa).

Alfalfa (*Medicago sativa* L.) is one of the most important forage plants because of its high nutritional quality, yield, and adaptability. Se is readily absorbed by alfalfa, which can also accumulate a certain amount of Se (Hu et al., 2022). Furthermore, Se-fortified alfalfa increased growth and survival rates, as well as promoted Se accumulation and colostrum antibodies in calves. Se-enriched feeds have grown to be a desirable resource for the preparation of useful feeds for animal breeding. To obtain Se supplementation safely and effectively, individuals commonly improve the Se content of meat, eggs, and milk in the human food chain via Se biofortification of alfalfa. Se is beneficial to plants on a physiological level (Kovács et al., 2021). However, the molecular mechanism underlying the impact of Se on the growth of alfalfa has not been thoroughly investigated.

Recent studies have demonstrated that non-coding RNAs (ncRNAs) play significant regulatory functions in various aspects of plant physiology, including growth, development, metabolism, and stress tolerance (Yu et al., 2019).

MicroRNAs (miRNAs) are a family of endogenous small ncRNA molecules that range in length from 20 to 24 nucleotides. These molecules can regulate post-transcriptional gene expression by either inhibiting the translation of target mRNAs or directing the cleavage of target mRNAs. Recent advancements in next-generation high-throughput sequencing technology and analytic approaches have enabled the identification of conserved and novel miRNAs in alfalfa (Tiwari et al., 2021a; Tiwari et al., 2022). A recent study has shown that alfalfa’s reactions to numerous environmental challenges (such as salt (Long et al., 2015) and freezing (Shu et al., 2016) are largely reliant on miRNA-related regulatory networks. The downregulation of the squamosa promoter binding protein-like (*SPL*) gene family, resulting from the overexpression of miR156 in transgenic alfalfa plants, had an impact on both the yield and quality parameters of the fodder (Aung et al., 2015). The regulatory impact of miRNA on the accumulation of nutrient-related metabolites may have an indirect effect on the quality of alfalfa. Long non-coding RNAs (lncRNAs) and circular RNAs (circRNAs) have recently been found in plants. LncRNAs are a type of ncRNA that exceed 200 nucleotides in length and possess the capacity to regulate gene expression through miRNA sponges or cis/trans-acting mechanisms (Chekanova, 2021). Endogenous circRNAs are generated via alternative circularization and possess a covalently closed structure (Lai et al., 2018). It has been proposed that lncRNAs, circRNAs, mRNAs, and pseudogenes can act as competing endogenous RNAs (ceRNAs) to competitively bind common miRNA response elements (*MREs*), thereby controlling a wide range of biological and developmental processes (Salmena et al., 2011; Paschoal et al., 2018). These ceRNAs are often referred to be miRNA sponges because they entrap specific miRNAs and decrease their activity. The flavor formation of oolong tea was studied using a complex ceRNA network composed of circRNA, mRNA, and miRNA (Zhu et al., 2020). The molecular pathways governing how ncRNAs select which genes to target during alfalfa growth are unknown; notably, the circRNA–miRNA–mRNA regulatory networks are involved in the development of growth and quality during Se fortification.

To investigate the effects of Se-induced stimulation on alfalfa biofortification, a solution containing 100 mg/kg of selenite was applied via spraying onto the leaves of the alfalfa plant (*M. sativa* L. cv. Kangsai). The effects of Se on alfalfa growth and quality improvement were determined by measuring agronomic factors and nutrients. The physiology and biochemistry of alfalfa revealed Se responses to oxidative stress. The influence of Se on the ultrastructure of alfalfa was confirmed using scanning electron microscopy (SEM). Furthermore, whole-transcriptome RNA sequencing (RNA-seq) of alfalfa was performed to determine the global molecular responses to Se treatment at the levels of both protein-coding RNAs (mRNAs) and non-coding RNAs (lncRNAs, miRNAs, and circRNAs). In addition, the ceRNA networks were established to provide additional insight into the regulatory mechanisms underlying the response of alfalfa to Se treatment. The findings offer information on the functions of ncRNA-mediated regulatory mechanisms in increasing the quality of alfalfa during Se biofortification.

## 2 Materials and methods

### 2.1 Plant materials

#### 2.1.1 Plant material and sample collection

The concentration of Se screened in the previous experiment at 0.75 kg/hm^2^ did not damage the growth of *M. sativa* L. cv. Kangsai and promoted it to a certain extent. Sodium selenite was sprayed evenly across the leaves of the alfalfa plant when its height reached 20 cm (on 28 August 2020). Se was then biofortified three times consecutively, once per week. The aboveground portion of the alfalfa plant was harvested, subjected to thorough washing, killed out for 30 min at 105°C, dried for 2 hours at 65°C, and weighed. Upper-second completely developed leaves were collected for the transcriptome and physio-biochemistry studies and stored at −80°C.

#### 2.1.2 Determination of Se content and agronomic traits

The above-mentioned dried materials were crushed and sieved through a 40-mesh sieve. Hydride generation atomic fluorescence spectrometry (HG-AFS) was used to determine the Se content (Chen et al., 2002). Hay production and plant height were calculated using the approach described in Hu et al. (2010). The Se accumulation was computed using the method of Hu et al. by multiplying the hay yield and the Se content of alfalfa harvested during the initial flowering period (Hu et al., 2010).

### 2.2 Nutritional quality analysis

Crude protein (CP), neutral detergent fiber (NDF), and acid detergent fiber (ADF) were measured after the dried sample was ground into powder according to (Schwanninger and Hinterstoisser, 2002). Relative feeding value (RFV) is a standard measure for evaluating the quality of forage that relies primarily on the concentrations of NDF and ADF. It has been extensively utilized in the United States as one of the most important methods for determining the quality of forage. RFV was determined by calculating the values of dry matter intake (DMI) and digestible dry matter (DDM) as follows (Zhao et al., 2021):


DMI(%BM) = 120/NDF (%DM)

DDM(%DM)=88.9−0.779×ADF(%DM)

RFV=DMI×DDM/1.29

### 2.3 Physiological analysis

Under refrigerated conditions, 0.5 g of alfalfa leaves was ground and extracted with 8 ml of a 50 mM sodium phosphate (pH 7.8) buffer solution. After being transferred to a centrifuge tube, the homogenate of plant tissue was centrifuged for 15 min at 12,000 *g* and 4°C. The obtained supernatant was then utilized to test the activity of glutathione peroxidase (GPX) and the concentration of (glutathione) GSH using a spectrophotometer and the protocols of Gill et al. (2015). Bradford’s method (Bradford, 1976) was used to measure the soluble protein, whereas Dubois’s phenol sulfuric acid technique (DuBois et al., 1956) was used to calculate the soluble sugar content. The estimation of MDA and H_2_O_2_ contents was carried out by Gill et al. (2015).

### 2.4 Photosynthesis analysis

The Fv/Fm was measured at 25°C using an IMAGING-PAM-MAXI instrument (Heinz Walz GmbH, Effeltrich, Germany) (Dong et al., 2021).

The photosynthetic rate (Pn) was measured utilizing a portable photosynthesis meter (LSPro-SD, ADC Bio-Scientific Ltd.UK).

### 2.5 *In situ* detection of ROS

*In situ* detection of ${\text{O}}_{2}^{-}$ and H_2_O_2_ was performed as described by Wu et al. (2015). The blue coloration observed in the experiment was attributed to the formation of formazan resulting from the reduction of NBT by ${\text{O}}_{2}^{-}$ . The polymerization process of DAB resulted in the apparent brown coloration of H_2_O_2_.

### 2.6 Ultra-structural observations

Transmission electron microscopy (TEM) was used to investigate cellular ultrastructure. TEM images were captured using a Hitachi HT7700 (Hu et al., 2014).

### 2.7 RNA extraction, library preparation, and RNA sequencing

From the three biological replicates for each group, six sRNA libraries (CK-1,2,3 and Low Se-1,2,3) were generated. Using Trizol reagent, total RNA was extracted and purified (Invitrogen, Carlsbad, CA, USA). The RNA concentration and purity of each sample were assessed using the NanoDrop ND-1000 instrument (NanoDrop, Wilmington, DE, USA). An Agilent 2100 with a RIN score greater than 7.0 was used to measure RNA integrity.

About 10 g of the total RNA was extracted as ribosomal RNAs for the lncRNAs, mRNAs, and circRNAs, and the remaining RNAs were then broken up into minute bits. The cleaved RNA fragments were subjected to reverse transcription using *E. coli* DNA polymerase I, RNase H, and dUTP, resulting in the production of cDNA. Subsequently, the aforementioned cDNA was employed in the construction of U-labeled second-stranded DNAs. The resultant cDNA collection had an average insert size of 300 bp (50 bp). Finally, the vendor-recommended procedure for performing paired-end sequencing on an Illumina Hiseq 4000 was implemented.

A small RNA sequencing library was generated using TruSeq Small RNA Sample Prep Kits (Illumina, San Diego, USA). Total RNA was ligated to the RNA 30 and RNA 50 adapters to produce cDNA constructs of the short RNAs. Reverse transcription was then performed, followed by PCR. Following that, the small cDNA fractions, ranging in length from 22 to 30 nt, were separated using 6% denaturing polyacrylamide gel electrophoresis. The library was then validated, and the cDNA constructs were purified. Then, single-end sequencing (50 bp) was performed using an Illumina Hiseq2500 at LC-BIO (Hangzhou, China) following the manufacturer’s suggested method.

### 2.8 Read mapping, transcriptome assembly, and differentially expressed mRNA

After eliminating the reads contaminated with adaptors (Martin, 2011), sequence quality was verified using FastQC.

Alfalfa’s genome was mapped using Bowtie2 (Langmead and Salzberg, 2012) and Hisat2 (Kim et al., 2015) (https://figshare.com/articles/dataset/genome_fasta_sequence _and_annotation_Files/12327602) (Li et al., 2020). The expression levels of all transcripts were estimated using StringTie (Pertea et al., 2015) and edgeR (Robinson et al., 2010) after the generation of the final transcriptome. StringTie (Pertea et al., 2015) was utilized to calculate mRNA expression levels using FPKM (Trapnell et al., 2010). The differentially expressed mRNAs were selected with log_2_ (fold change) >1 or log_2_ (fold change)<−1 and with statistical significance (*p* value< 0.05) by R package–edgeR (Robinson et al., 2010).

### 2.9 Identification of lncRNAs and prediction of their target genes

After reading assembly with StringTie, the known mRNA, transcripts with a fragment count of under three, transcripts shorter than 200 nt, and open reading frames (ORFs) longer than 300 nt were removed, and the remaining transcripts were identified for lncRNA. If these remaining transcripts have the potential to encode proteins, they are classified as novel mRNA, defined as mRNA, and filtered. The filtered transcripts were screened as novel lncRNAs according to the conditions of CPC score< −1 and CNCI score< 0 by the software CPC (coding potential calculator) (Kong et al., 2007) and CNCI (coding non-coding index) (Sun et al., 2013). The FRKM and differential expression analysis of lncRNAs were computed using the same methodology as for mRNAs (Robinson et al., 2010). To examine the function of lncRNAs in Se biofortification, potential cis- and trans-genes of differentially expressed lncRNAs (DELncRNAs) were projected according to chromosome position. The target genes within a 10-kb frame upstream or downstream of the lncRNAs were investigated (Liao et al., 2011). Functional analysis of these genes was performed using the BLAST2GO (Conesa et al., 2005).

### 2.10 Identification and analysis of circRNAs

In this investigation, the CIRCexplorer and CircRNA Identifier (CIRI) tools were used to identify candidate circRNAs. Using CIRCexplorer and a TopHat-Fusion (v2.1.0) mapping strategy, the unmapped reads were processed to generate a database of fusion transcripts (Zhang et al., 2014). After the assembled linear RNA transcripts were aligned with CIRI, back-spliced junction reads≥ 2 were identified and removed from further analysis using a computational workflow (Gao et al., 2015). For the subsequent analysis, the overlapping outputs of CIRCexplorer and CIRI were utilized. Fragment Per Kilobase of Exon Per Million Mapped Fragments (FPKM) data were calculated using the StringTie method to quantify circRNA expression (Trapnell et al., 2010). log_2_FC > 1 and *p<* 0.05 were confirmed as differentially expressed circRNAs (DEcircRNAs).

### 2.11 Identification and analysis of miRNA

Prior to undergoing processing with the internal tool ACGT101-miR (LC Sciences, Houston, TX, United States), the raw sequencing reads were subjected to the Illumina pipeline filter (Solexa 0.3) (Ma et al., 2018). To attain accurate data, the initial raw data underwent a quality control process that involved the removal of 3′ joints and unnecessary sequences. For further analysis, reads with a base length of 18–25 nt are set aside. The adapter dimers, low complexity reads, common RNA families (rRNA, tRNA, snRNA, and snoRNA), and repeats were later extracted from the retained read and stored as clean short RNA reads in the NCBI GenBank, Repbase, and Rfam databases, respectively (http://www.sanger.ac.uk/software/Rfam). According to the method of Tiwari et al. (2021b), miRNAs in alfalfa were predicted. The folded RNA secondary structures that fulfill all the latest criteria for miRNA annotation as described in Axtell and Meyers (2018) were chosen for mature miRNA prediction. Before analyzing the count of differentially expressed sequences, normalization of miRNA expression levels was performed using global normalization techniques (Huber et al., 2002). The utilization of online web service IDEG6 (http://compgen.bio.unipd.it/bioinfo/software/) was employed for this purpose. The differentially expressed miRNAs were used to calculate the differentially expressed sequence count (DEmiRNAs) when the absolute log_2_FC > 1 and *p<* 0.05. The psRobot (v1.2) software (Wu et al., 2012) was used to predict the target genes for DEmiRNAs.

### 2.12 Construction and analysis of ceRNA regulatory network

CeRNA regulatory networks were constructed to acquire the interaction of lncRNAs, circRNAs, miRNAs, and mRNAs. The miRNA–mRNA pairings were predicted using Targetfinder (Fahlgren and Carrington, 2010). The prediction result was evaluated based on the Alignment score, with a maximum penalty of four points. The lower the score, the more complete and credible the match. The prediction of miRNA–lncRNA/circRNA interactions was conducted through the utilization of Ssearch36 (Pearson, 2016) from the FASTA3 program package, in conjunction with Perl scripts, following the principles outlined by Sun et al. (2020). The interaction networks were visualized using Cytoscape (version 3.8) software.

### 2.13 qRT-PCR validation of DEmRNAs, DElncRNAs, DEcircRNAs, and DEmiRNAs

The expression levels of DEmRNAs, DElncRNAs, and DEmiRNAs were verified by qRT-PCR. The *18S* rRNA gene was selected as the reference gene for normalizing DEmRNAs, DElncRNAs, and DEcircRNAs (Fu et al., 2014). Normalization of DEmiRNA expression levels was performed using the CT values obtained for the *U6* gene (Luo et al., 2018). The quantification of expression level was performed using the 2^−ΔΔCt^ method. All qRT-PCR tests were performed three times in biological replicates and three times in technical replicates. The comprehensive list of primers can be found in **Additional file 1**: **Table S1**.

## 3 Results

### 3.1 Effects of foliar spraying Na_2_SeO_3_ on Se accumulation and growth of alfalfa

**Figure 1:**
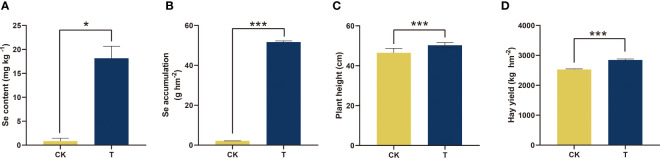
Se accumulation and agronomic characteristics of alfalfa treated with selenite. **(A)** Se content; **(B)** Se accumulation; **(C)** plant height; **(D)** hay yield. The experiments and statistical analyses were performed in three biological replicates. *Significant difference (*p<* 0.05); ***extremely significant (*p<* 0.001).

To evaluate the absorption and accumulation of Se by alfalfa, the Se content and accumulation were initially measured (**Figure 1**). Se treatment significantly increased Se content (*p<* 0.05) and Se accumulation (*p<* 0.001) in alfalfa. The content and accumulation of Se increased by 21 and 24 times, respectively (**Figures 1A, B**). Furthermore, the application of Se treatment exhibited an impact on the agronomic characteristics of alfalfa, encompassing plant height and hay yield. In comparison to the control group, the application of Se to alfalfa resulted in a 5.13% increase in plant height and a 6.61% increase in hay yield, as depicted in **Figures 1C, D**.

### 3.2 Effects of Na_2_SeO_3_ on nutrient and quality of alfalfa

**Figure 2:**
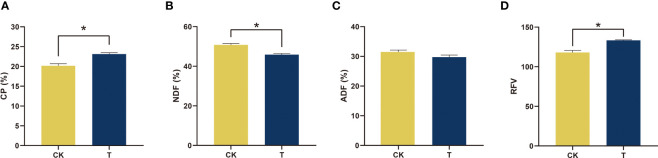
Nutrients and quality of alfalfa treated with selenite. **(A)** CP; **(B)** NDF; **(C)** ADF; **(D)** RFV. The experiments and statistical analyses were performed in three biological replicates. *Significant difference (*p<* 0.05).

Crude protein (CP) was one of the most essential indices used to determine the quality of alfalfa, and there was a significant difference between the control and treatment groups. It is crucial for the nutritional quality of alfalfa. The application of Se treatment resulted in a statistically significant increase (*p<* 0.05) in CP levels compared to the control group. The CP levels reached 23.12% (DM), as illustrated in **Figure 2A**. Upon administration of a suitable quantity of Se, the neutral detergent fiber (NDF) and acid detergent fiber (ADF) experienced a reduction of 9.69% and 5.52% (DM), respectively, in comparison to the control, as depicted in **Figures 2B, C**. The quality was assessed based on the relative feeding value (RFV), and it was observed that the RFV increased significantly (*p<* 0.05) by 13.06% to 133 with the addition of 100 mg/kg Se, as depicted in **Figure 2D**.

### 3.3 Effects of Na_2_SeO_3_ on the physiology and biochemistry of alfalfa

**Figure 3:**
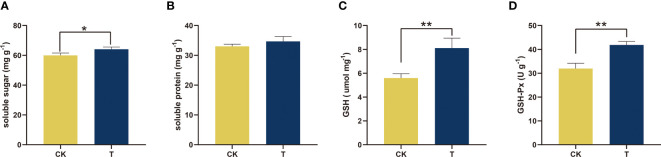
Physiological and biochemical traits of alfalfa treated with selenite. **(A)** Soluble sugar; **(B)** soluble protein; **(C)** GSH; **(D)** GSH-PX. The experiments and statistical analyses were performed in three biological replicates. *Significant difference (*p<* 0.05); **highly significant difference (*p<* 0.01).

**Figure 4:**
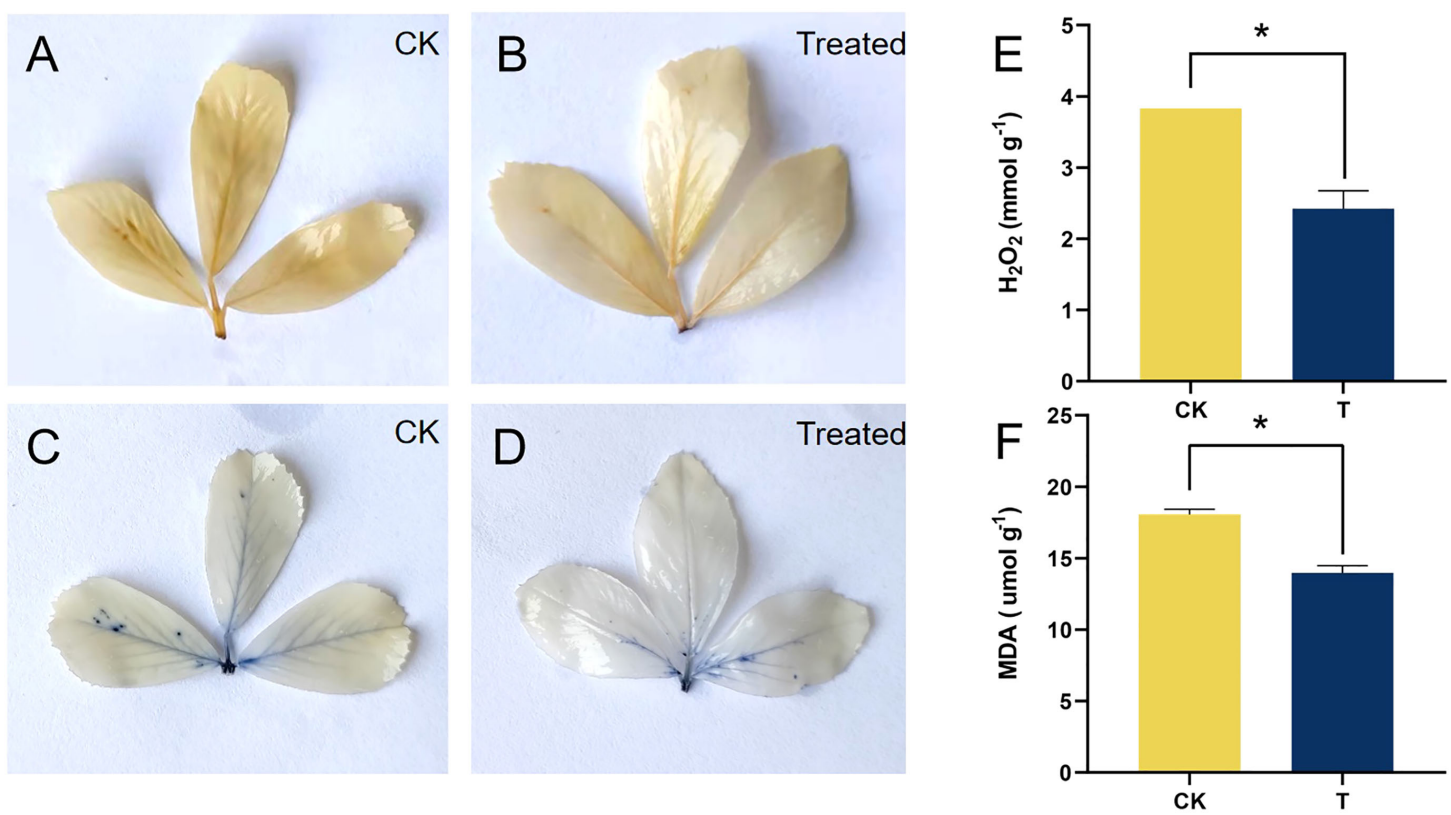
Qualitative and quantitative analysis of ROS of alfalfa treated with selenite. NBT and DAB staining of alfalfa leaves under control (above) and Se treatment (below), with brown aggregation products representing H_2_O_2_ **(A**, **B)** and blue aggregation products representing ${\text{O}}_{2}^{-}$ **(C, D)**. **(E)** Determination of H_2_O_2_ content. **(F)** Determination of MDA content. *Significant difference (*P* < 0.05).

**Figure 5:**
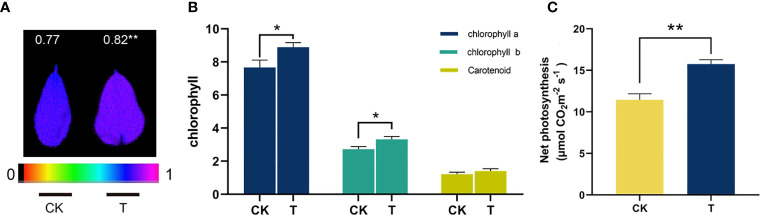
The photosynthesis-related characteristics of selenite-treated alfalfa. **(A)** Fv/Fm; **(B)** the contents of chlorophyll a, chlorophyll b, and carotenoids; **(C)** net photosynthesis. The experiments and statistical analyses were performed in three biological replicates. *Significant difference (*p<* 0.05); **highly significant difference (*p<* 0.01).

The physiological status was assessed to gain a deeper comprehension of the impact of Se on the growth of alfalfa (**Figures 3**–**5**). The soluble sugar and protein levels, which are fundamental metabolic and crucial osmoregulatory substances, were assessed in the context of Se treatment. The results indicated a significant increase in both parameters, as illustrated in **Figures 3A, B**. Se (100 mg/kg) considerably (*p<* 0.05) raised soluble sugar levels by 6.88% in comparison to the control (**Figure 3A**).

S may lead to oxidative stress and the production of ROS. When compared to the control, the 100 mg/kg Se treatment considerably (*p<* 0.05) lowered the levels of H_2_O_2_ and MDA by 36.72% and 22.63%, respectively (**Figures 4E, F**). In the qualitative staining experiment involving ROS, it was observed that the brown polymerization products of H_2_O_2_ and blue polymerization products of ${\text{O}}_{2}^{-}$ exhibited a significant decrease at a Se treatment of 100 mg/kg, as compared to the control (**Figures 4A, B**). These results are consistent with the quantitative results of H_2_O_2_ after Se treatment.

To compare the effects of Se-treated alfalfa versus untreated alfalfa on the endogenous antioxidant system, GSH-Px and GSH levels were measured in relation to antioxidant capacity. The utilization of Se exhibited a noteworthy enhancement in the antioxidant capacity in comparison to the control (*p<* 0.01). As compared to the control, 100 mg/kg Se increased the GSH content and GSH-Px activity by 44.82% and 31.10%, respectively (**Figures 3C, D**).

To clarify the mechanisms by which Se promotes photosynthesis in alfalfa, an investigation was conducted to analyze alterations in chlorophyll content, chlorophyll fluorescence, and net photosynthesis (**Figure 5**). Chlorophylls are essential pigments with numerous functions, as they regulate the amount of photosynthetically active radiation absorbed during photosynthesis (Feng et al., 2020). There was a discernible enhancement observed in the levels of the three photosynthetic pigments. The administration of 100 mg/kg Se resulted in a significant increase in the levels of chlorophyll a, chlorophyll b, and carotenoids by 15.91%, 21.67%, and 15.31%, respectively (**Figure 5B**). Maximum light efficiency of PSII, as measured by Fv/Fm, was found to be improved by 6.49% after Se treatment (**Figure 5A**). Furthermore, it was observed that the net photosynthesis of alfalfa exhibited a statistically significant increase (*p<* 0.05) of 37.59% when subjected to reduced concentrations of Se (**Figure 5C**).

Consequently, the application of 100 mg/kg of spray on alfalfa resulted in a positive impact on its growth and development. This was attributed to the maintenance of redox balance and reduced levels of ROS, which, in turn, facilitated the improvement of photosynthesis.

### 3.4 Effects of Na_2_SeO_3_ on the ultrastructure of alfalfa

**Figure 6:**
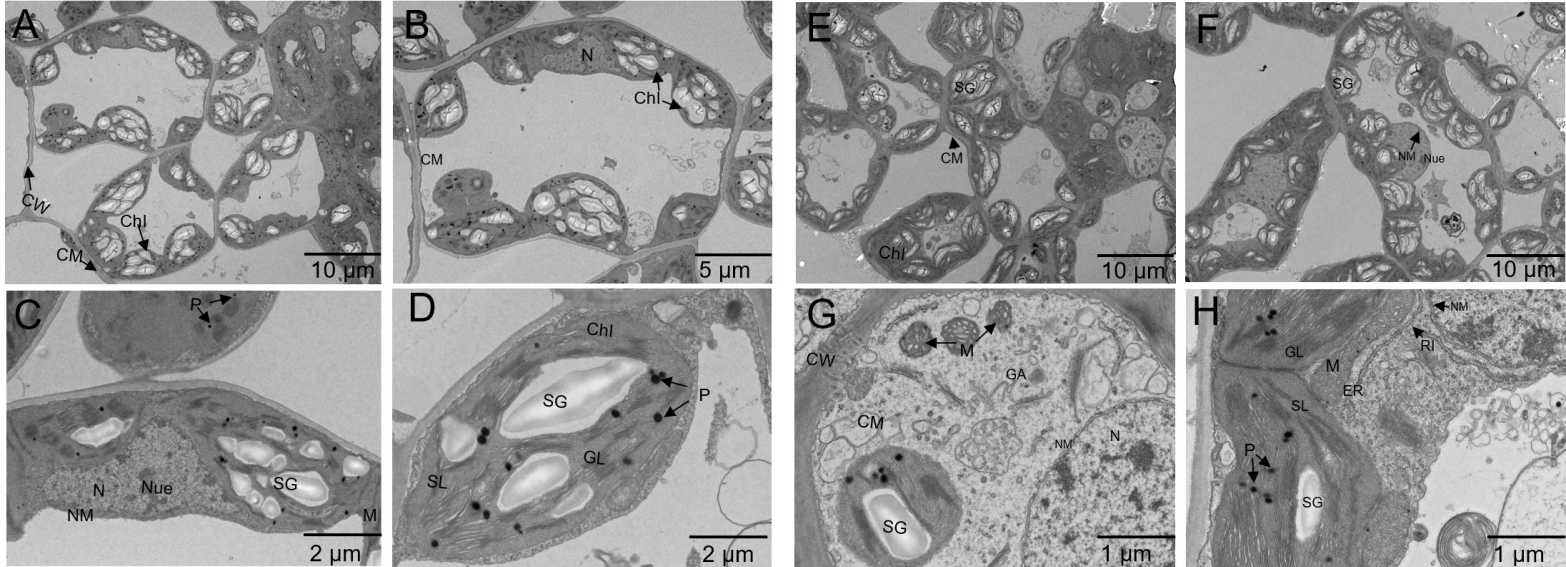
Ultrastructure of alfalfa mesophyll cells treated with selenite: control conditions **(A–D)**; 100 mg/kg Se treatment **(E–H)** [cell wall (CW), plasma membrane (CM), nucleus (N), nucleoli (Nue), nuclear membrane (NM), mitochondria (M), chloroplast (Chl), grana lamellae (GL), stroma lamellae (SL), starch granules (SG), plastid pellets (also known as osmiophilic granules, P), endoplasmic reticulum (ER), Golgi apparatus (GA), ribosome (RI)].

TEM, the ultrastructure of alfalfa mesophyll cells was studied. Se treatment led to a distinct extent of subcellular structure compared to the control (**Figure 6**). Under controlled conditions, the mesophyll cells of alfalfa exhibited a smooth cell wall, a distinct plasma membrane boundary, a well-developed nucleus, and smooth nucleoli, along with a complete nuclear membrane. There were clear mitochondria (though relatively small) and well-formed chloroplasts with a regular arrangement of thylakoids (**Figures 6A–D**). In contrast, at a Se treatment of 100 mg/kg, the cell walls exhibited clarity and thickness, accompanied by a well-organized cytoplasmic membrane. The Golgi, endoplasmic reticulum, and ribosomal organelles have demonstrated exuberant intracellular metabolism. The mitochondria exhibited a mature phenotype and were densely distributed throughout the cells. The chloroplasts were well-developed, large, and many, with complete and smooth membranes. The thylakoids were aligned in parallel along the chloroplast’s long axis, and the grana lamella structure was evident and closely arranged. Furthermore, when compared to the control group, the administration of 100 mg/kg Se resulted in an increase in starch grains within the chloroplasts, which were distributed along the long axis of the middle part of the chloroplast (**Figures 6E–H**).

### 3.5 Global responses of miRNA to Na_2_SeO_3_ treatment

**Figure 7:**
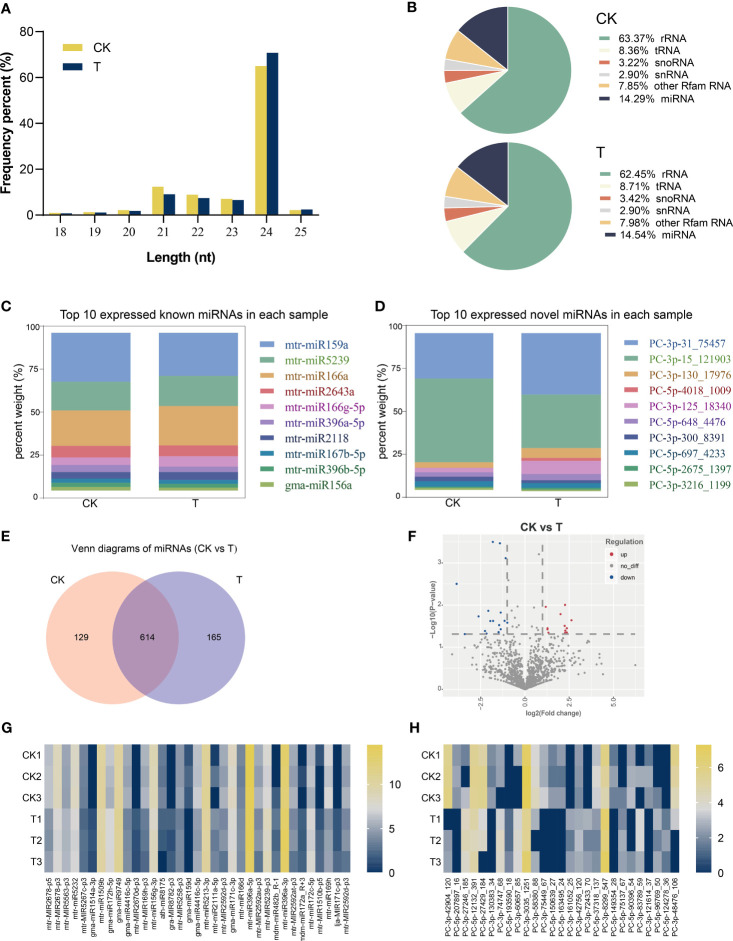
Identification and analysis of differentially expressed miRNAs (DEmiRNAs) under Se treatment. Length distribution of all identified small RNAs **(A)**. Percentage of different types of small RNAs **(B)**. Top 10 expressed known **(C)** and novel **(D)** miRNAs in each sample.; Venn diagram showing the detected miRNAs **(E)**; DEmiRNAs in Control and treated with Se **(F)**. Heat map of all known **(G)** and novel **(H)** DEmiRNAs.

The control and Se-treated small RNA libraries produced 10,195,291 and 12,632,817 raw reads, respectively. Following the removal of adaptor sequences, low-quality sequences, and reads that did not meet the length criteria of 18 nt to 32 nt, the resulting dataset comprised 3,950,251 and 5,264,445 high-quality reads. The majority of clean reads exhibited a length of 20–25 nt (**Figure 7A**). The results of small RNA classification exhibited minor variations across diverse treatments, with rRNA accounting for 63%, tRNA for 8%, miRNA for 14%, and the remaining 15% comprising other types, as illustrated in **Figure 7B**. A total of 1,234 known and 1,285 novel miRNAs were identified from approximately 14% of clean reads (**Additional file 2**: **Table S2**). **Figures 7C, D** depict the top 10 miRNAs expressed in each sample, with mtr-miR159a and PC-3p-31_75457 displaying the highest expression levels. It was determined that a total of 614 miRNAs were expressed in both the Control and Se treatments (**Figure 7E**). Differentially expressed miRNAs (DEmiRNAs) were screened according to the multiples of expression > 2 and *p*< 0.05. A total of 27 upregulated and 35 downregulated DEmiRNAs were identified in Control and Se-treated groups, respectively (**Figure 7F**; **Additional file 3**: **Table S3**). The typical expression patterns of these DEmiRNAs are displayed in **Figures 7G, H**. Significant variations in their expression levels were observed between CK and Se-treated alfalfa.

### 3.6 Global responses of lncRNA and circRNA to Na_2_SeO_3_ treatment

**Figure 8:**
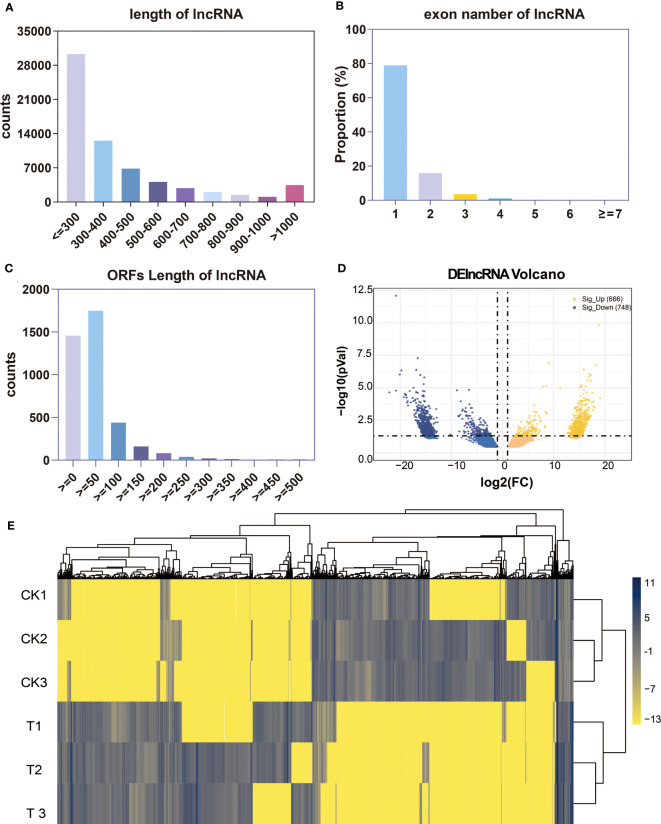
Identification and analysis of differentially expressed lncRNAs (DElncRNAs) under Se treatment. Detected lncRNAs on the transcript length **(A)**, exon number **(B)**, ORF length and FPKM value **(C)**. Venn diagram showing the number of DElncRNAs in alfalfa **(D)**; heat map of all DElncRNAs **(E)**.

After the elimination of the identified mRNA and transcripts with a length of less than 200 bp, the process of lncRNA prediction was carried out on the residual transcripts. Under the filtering conditions of CPC (Coding Potential Calculator) Filter CPCFilter (score ≤ 0.5) and CNCI (Coding-Non-Coding Index) Filter [(length ≥ 200) and (exon ≥ 1) and (score ≤ 0)], a total of 112,230 lncRNAs were finally obtained in alfalfa. The analysis of genomic characteristics of lncRNAs revealed that a considerable proportion of lncRNAs had long transcripts (<300 bp). Furthermore, a higher percentage of lncRNAs were observed to have one to three exons. Additionally, the ORF length of lncRNAs was predominantly distributed in the range of 0–100, as depicted in **Figures 8A–C**. A total of 1,414 DElncRNAs were detected in the Control and Se-treated groups, with 666 upregulated and 748 downregulated, using a cutoff of log_2_FC > 1 and *p<* 0.05 (**Figure 8D**; **Additional file 4**: **Table S4**). In alfalfa, the FPKM values of DElncRNAs exhibited a range of −13 to 11. The general expression profiles of DElncRNAs are shown in **Figure 8E**. The DElncRNAs in the Se-treated and CK groups were clustered independently, whereas their three repeats were grouped.

**Figure 9:**
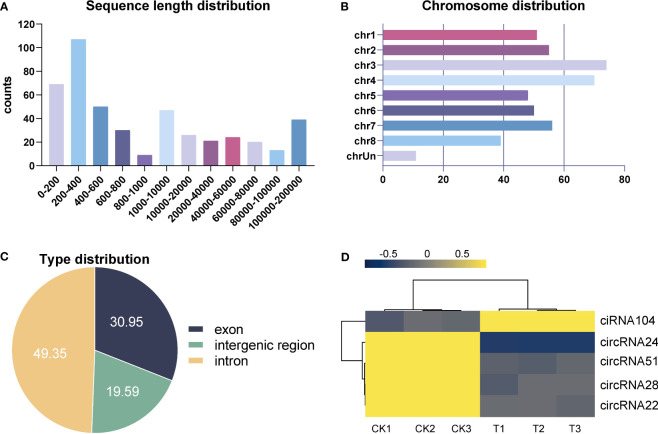
Identification and analysis of differentially expressed circRNAs (DEcircRNAs) under Se treatment. Sequence length, chromosome, and type distribution of all identified circRNAs **(A–C)**. Heat map of all DEcircRNAs **(D)**.

A total of 454 circRNAs were detected in the leaf of alfalfa. Among them, 49.35%, 30.95%, and 19.59% were classified as intergenic, exon, and intron types, respectively, as illustrated in **Figure 9C**. **Figure 9A** displays the distribution of sequence length for circRNAs, indicating that the majority of circRNAs ranged from 200 bp to 400 bp. Furthermore, the highest number of circRNAs were observed in Chromosome 3 (chr3), followed by chr4, chr7, and chr2, as illustrated in **Figure 9B**. Five DElncRNAs (one upregulated and four downregulated) were identified in the control and Se-treated groups with an absolute value of log_2_FC greater than 1 and a *p*-value less than 0.05. Refer to **Additional file 5**: **Table S5** for further details. The expression profiles of DEcircRNAs in each group were visualized using a heat map, which revealed that the majority of DEcircRNAs exhibited elevated expression levels in the control group (**Figure 9D**). These results suggest that the circRNAs also play a role in the responses to Se treatment.

### 3.7 CeRNA regulatory network in response to Na_2_SeO_3_ treatment

**Figure 10:**
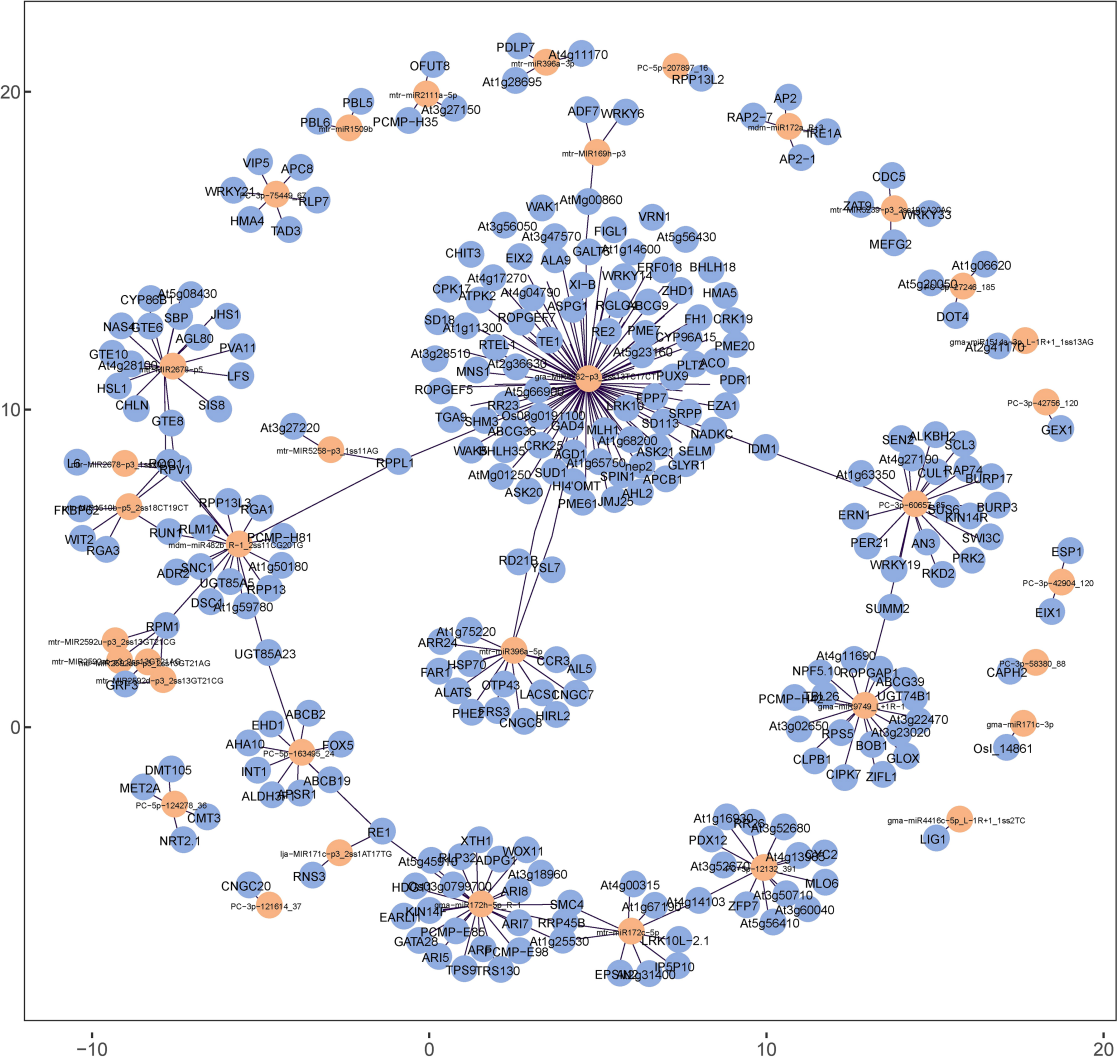
Regulatory network constructed with targeted mRNA (yellow) and DEmiRNAs (blue) in alfalfa.

The miRNA–mRNA regulatory network analysis was conducted as the initial step to reveal the global regulatory network of protein-coding RNAs and ncRNAs in response to Se treatment. Based on perfect or nearly perfect complementarity between miRNAs and their target mRNAs, 288 mRNAs were predicted as potential DEmiRNA targets. The targeted mRNAs of the DEmiRNAs are presented in **Table S6** of **Additional file 6** and illustrated in **Figure 10**. The study revealed that a significant proportion of DEmiRNAs in alfalfa, precisely 88.7% (55 out of 62), exhibit the ability to target multiple mRNAs.

**Figure 11:**
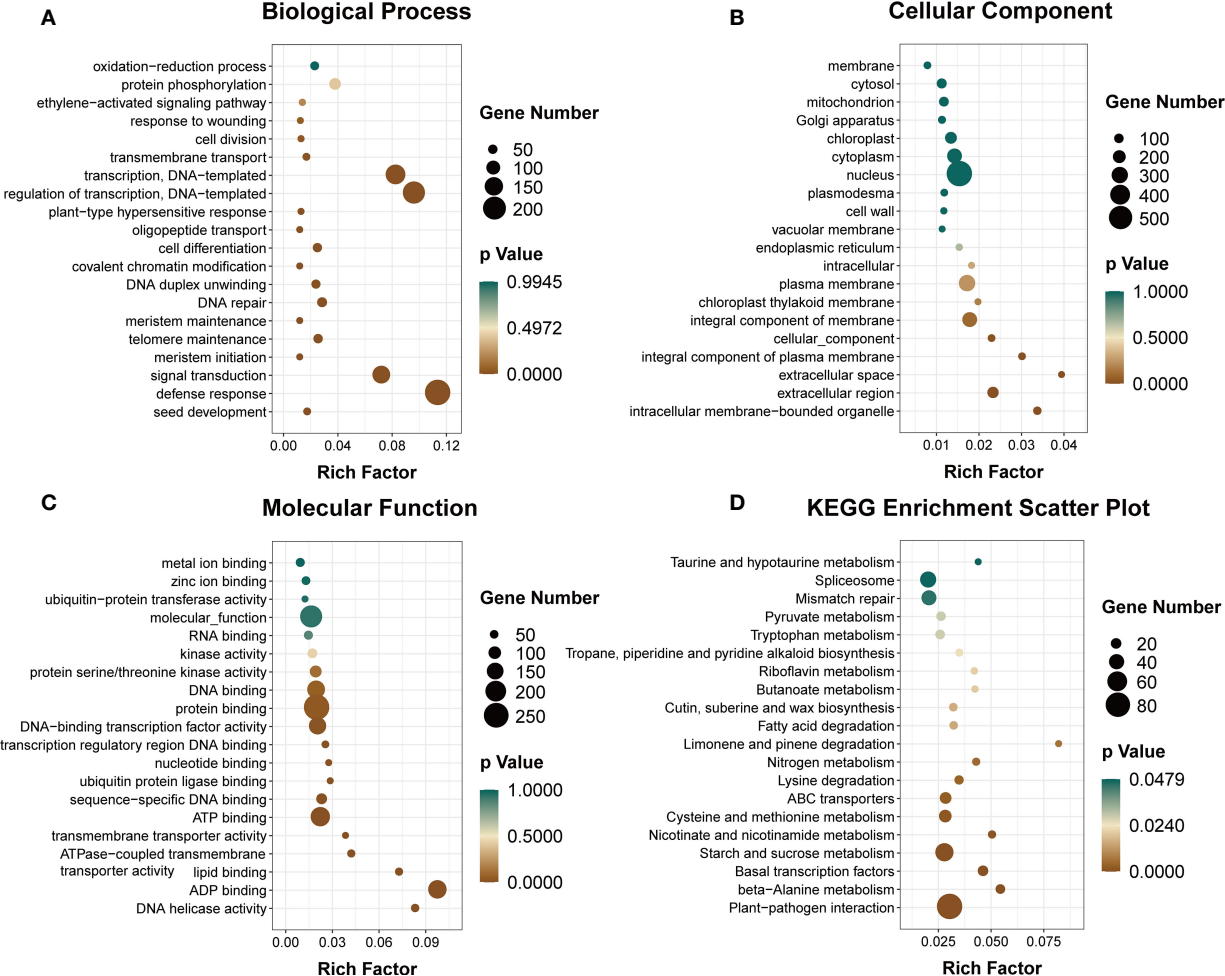
Functional analyses of targets of DEmiRNAs in alfalfa. Analysis of GO enrichment **(A–C)** and KEGG pathways **(D)**.

The GO annotation analysis of DEmiRNA targets in alfalfa revealed that a majority of them were associated with various biological processes such as defense response (GO:0006952), transcriptional regulation (GO:0006355), signal transduction (GO:0007165), DNA repair (GO:0006281), and oxidation–reduction processes (GO:0055114) under the category of biological processes (BP). Additionally, these targets were annotated to various cellular components (CC) such as the nucleus (GO:0005634), plasma membrane (GO:0005886), cytoplasm (GO:0005737), and chloroplast (GO:0009507). Furthermore, the molecular function (MF) analysis revealed that these targets were associated with protein binding (GO:0005515), ATP binding (GO:0005524), and DNA-binding transcription factor activity (GO:0003700) (**Figures 11A–C**; **Additional file 8**: **Table S8**).

The results of the KEGG pathway analysis indicated that the targets of the DEmiRNAs were associated with 95 pathways. Among these pathways, the 20 most significantly enriched KEGG pathways were identified and are presented in **Figure 11D** and **Additional file 8**: **Table S8**. These pathways include Plant–pathogen interaction (ko04626), Basal transcription factors (ko03022), Starch and sucrose metabolism (ko00500), Cysteine and methionine metabolism (ko00270), ABC transporters (ko02010), and Nitrogen metabolism (ko00910), among others.

**Figure 12:**
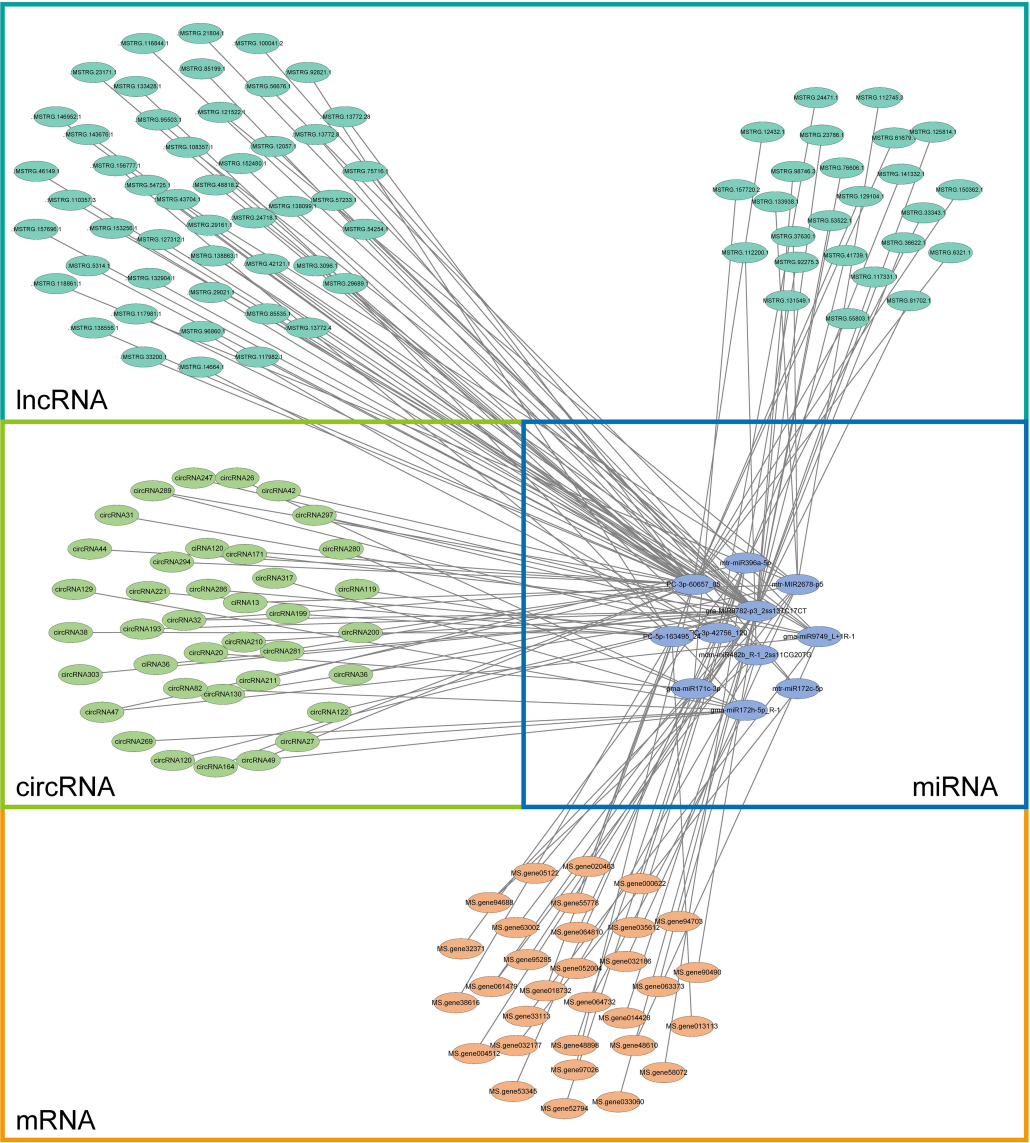
CeRNA network constructed with targeted mRNAs (blue) of all DEmiRNAs (yellow), DElncRNAs (cyan–blue), and DEcircRNAs (green) in alfalfa.

According to the theory of competing endogenous RNA (ceRNA), lncRNA/circRNA can interact with miRNA and release mRNA from miRNA binding. Targeted mRNAs, DEmiRNAs, DElncRNAs, and DEcircRNAs from alfalfa were utilized to construct a ceRNA network (**Figure 12**; **Additional file 9**: **Table S9**).

### 3.8 Validation of gene expression by qRT-PCR

**Figure 13:**
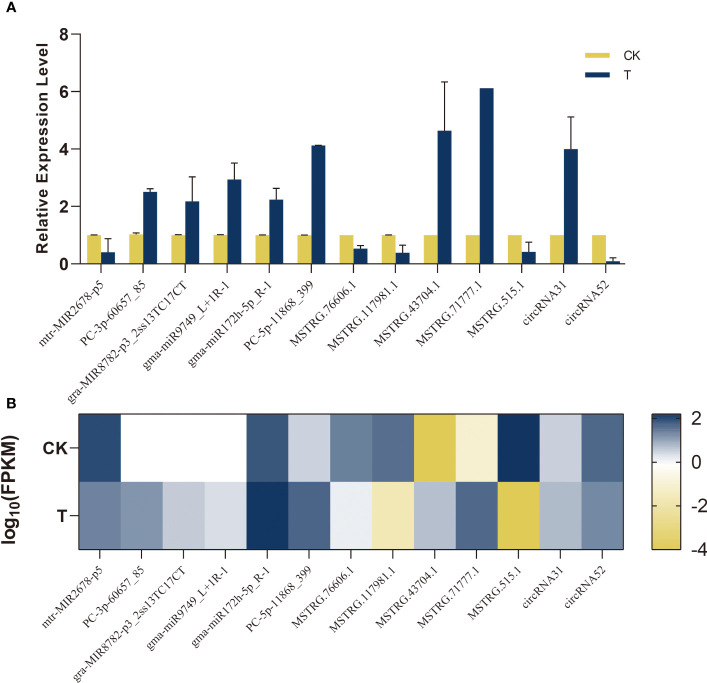
Validation of sequencing data by qRT-PCR. Bar graphs indicate relative expression level using qRT-PCR **(A)**; heat map indicates FPKM values transformed log_10_ based on RNA-seq **(B)**.

To assess the precision of RNA-seq in Na_2_SeO_3_-treated leaves compared to the control, a limited number of miRNAs, lncRNAs, and circRNAs were randomly chosen and validated through qRT-PCR (**Figure 13**). The qRT-PCR results indicated that the expression patterns of six selected miRNAs, consisting of five upregulated and one downregulated miRNA, were consistent with our sequencing data. The expression of MSTRG.43704.1 and MSTRG.71777.1 was upregulated compared to the control. It was confirmed that the expression levels of the remaining lncRNAs were downregulated. There was an agreement between the RNA levels and the computed FPKM for two randomly selected circRNAs (designated circRNA31 and circRNA52) that were quantified using divergent and convergent primer sets. The present study provides evidence that the expression patterns of miRNA, lncRNA, and circRNA are reliable indicators for investigating the transcriptional changes induced by Se in alfalfa.

## 4 Discussion

Se is not required for the survival of plants, whereas it is important for animals. The primary source of the vital nutrient Se for humans is derived from plant-based sources. To mitigate the adverse health effects of insufficient dietary Se, several food items have been enriched with Se. Additionally, it is suggested that the biofortification of crops like alfalfa can be achieved by supplementing Se through fertilizers (Hu et al., 2022). There are currently very few preliminary studies on Se enrichment in alfalfa, with the majority of research focusing on the kinetics of selenium uptake in multiple-harvested hay (Kovács et al., 2021), the impact of Se on quality and yield (Hu et al., 2010), and the effect of animal husbandry after feeding Se-enriched alfalfa (Pan et al., 2021). Currently, high-throughput sequencing is an effective method for analyzing gene expression profiles and identifying low-abundance ncRNAs (Li et al., 2014; Zhang et al., 2018). This study provided a comprehensive outlook on the regulatory function of ncRNAs in the Se treatment of alfalfa. The integration of a network of lncRNA/circRNA–miRNA–mRNA interactions was achieved through the utilization of throughput screening and bioinformatics analysis.

### 4.1 miRNA is a key regulator during Se biofortification of alfalfa

Over the recent decades, several studies have verified that Se can trigger the defense mechanism of plants and promote their growth. In a recent report, it was indicated that reduced Se levels were found to enhance the growth of alfalfa in both normal and abiotic stress conditions. The exact gene expression pattern of the plant in response to Se remains uncertain. This study aimed to investigate potential metabolic regulatory mechanisms in plants exposed to Se through a combined analysis of miRNA target mRNAs and miRNA expression profiles. The objective was to identify both known and novel genes of alfalfa in response to Se. The levels of miRNA transcripts in alfalfa, comprising 35 established miRNAs and 27 novel miRNAs, were significantly impacted by the application of Se. The application of Se had a significant impact on gma-miR172h-5p_R-1, mtr-miR156g-3p_1ss3TC, gma-miR9749_L+1R-1, and mtr-miR5213-3p_1ss8CT. The majority of the miRNAs that were anticipated to target genes were observed to exert their influence on numerous target genes. The 288 target genes are primarily associated with various pathways such as basal transcription factors (ko03022), starch and sucrose metabolism (ko00500), DNA replication (ko03030), cysteine and methionine metabolism (ko00270), and protein processing in the endoplasmic reticulum (ko04141), among others. It has been reported that miR172 is a positive regulator of salt tolerance in rice and wheat by maintaining ROS homeostasis (Cheng et al., 2021). Furthermore, adequate levels of miR156 transcripts are crucial in promoting drought tolerance in alfalfa (Feyissa et al., 2019). To summarize, these significantly impacted miRNAs in this study may be implicated in Se-induced plant growth regulation.

### 4.2 Involvement of miRNAs in Se biofortification of alfalfa by targeting transcription factors

Transcription factors (TFs) in higher plants play a significant role in various growth and development processes such as signal transduction, cellular morphogenesis, and response to environmental stressors. These TFs have a direct impact on the agronomic and financial aspects of crops. Higher plant TFs are encoded by approximately 10% of genes (Zhang et al., 2012). Li et al. (2021) conducted a comprehensive analysis to identify regulatory protein genes and miRNAs that respond to Se stimulus in *Pueraria lobata* (wild.) Ohwi. The identified genes were found to regulate sulfate and phosphate transporters, ROS scavenging, and isoflavone metabolism in the plant. The present investigation revealed that a total of 34 mRNAs were identified as encoding TFs, which were subjected to targeting by 14 DEmiRNAs. The gene MS.gene 067454, which encodes a *bHLH35* transcription factor, was subjected to targeting by gra-MIR8782-p3_2ss13TC17CT. It is noteworthy that a single miRNA has the potential to modulate the activity of several TFs, while conversely, multiple miRNAs may exert regulatory control over a single TF. For example, gra-MIR8782-p3_2ss13TC17CT was identified as a potential regulator of four TFs, namely, *bHLH25* (bHLH family), *WRKY21* (WRKY family), *ERF018* (ethylene-responsive family), and *RAP2-4* (ethylene-responsive family). The *AP2* TF was expected to be regulated by four miRNAs, including gra-MIR8782-p3_2ss13TC17CT, mdm-miR172a_R+3, gra-MIR8782-p3_2ss13TC17CT, and mtr-miR396a-5p. *ERF96* (Ethylene response TF family) is involved in the control of selenite tolerance, and overexpression of *ERF96* increased selenite resistance in Arabidopsis (Jiang et al., 2020). The *WRKY* TFs represent a substantial group of regulatory proteins that play a crucial role in the developmental mechanisms of plants as well as their reactions to various biotic and abiotic stimuli. *WRKY* gene plays an important role in maintaining Se homeostasis and tolerance in *Arabidopsis thaliana* (Wu et al., 2020). *WRKY* transcription factor 31 is controlled by mtr-MIR169h-p3 and may be involved in the maintenance of Se homeostasis in alfalfa. These findings revealed that TFs and miRNA are key gene regulators that exhibit close coordination in their response to Se treatment in alfalfa.

### 4.3 Involvement of miRNAs in Se biofortification of alfalfa by targeting photosynthesis, carbohydrate metabolism, and protein processing

Previous research has indicated that Se is advantageous to the development and overall health of certain plant species (Schiavon et al., 2016; Cabral Gouveia et al., 2020). The impact of Se on the growth of alfalfa is currently insufficiently understood. Organic forms of Se are primarily utilized by plants (Sors et al., 2005; White, 2018). The promotion of antioxidant enzyme activities, such as GPX, and antioxidant contents, such as GSH, was observed at a concentration of 100 mg/kg in alfalfa (**Figure 3**). The results of both qualitative and quantitative experiments indicate that the application of 100 mg/kg Se treatment resulted in the maintenance of low levels of plant stress markers, namely, MDA and H_2_O_2_ (as shown in **Figure 4**). The results indicate that alfalfa exhibited a greater antioxidant capacity at a concentration of 100 mg/kg, which is in line with previous research conducted on *Brassica napus* (Ulhassan et al., 2019). Furthermore, the observation of higher levels of soluble proteins and sugars at a concentration of 100 mg/kg in alfalfa suggests that it possesses a greater capacity to synthesize these osmotic substances in response to Se-phytotoxicity (**Figure 3**). This finding is consistent with previous research conducted on *Neptunia amplexicaulis* (White, 2016). These findings revealed that Se plays a crucial role in plant growth and stress resistance. Photosynthesis is a critical metabolic process in plants that provides the primary energy source for plant growth. The increase in chlorophyll content provided the basis for photosynthesis at 100 mg/kg (**Figure 5B**). This observation is in agreement with the findings of Mostofa et al. (2017) in their study on rice, which was induced by Se. Furthermore, the notable increase of Fv/Fm demonstrated the amplification of light energy absorption by antenna proteins and energy distribution-transfer between PS II and PS I in alfalfa as a result of Se treatment, as illustrated in **Figure 5A**. The aforementioned factors have been demonstrated to directly or indirectly enhance the photosynthesis of alfalfa under 100 mg/kg Se treatment, as evidenced by the net photosynthesis measurements obtained through the use of a portable photosynthesis meter (**Figure 5C**). According to the biomass analysis (**Figure 1D**), Se could greatly improve plant growth and increase the accumulation of organics (crude protein) (**Figure 2A**). These findings present very compelling evidence for the growth-promoting influence that Se has on plants when grown under field conditions. The addition of moderate amounts of Se to alfalfa has been found to increase its antioxidant activity and regulate the levels of carbohydrates and proteins, thereby creating a conducive environment for nutrient accumulation *in vivo*.

Transmission electron microscopy is a commonly employed technique in the field of plant microstructure analysis to observe subcellular structures (Kiyoto et al., 2022). According to Ulhassan et al. (2019), variations in Se concentrations significantly altered the ultrastructure of cells in Oilseed rape. At 100 mg/kg, there was a defined and thick cell wall, a smooth nucleus, and more mature organelles like chloroplasts and mitochondria. The effects of Se treatment on the ultrastructure of alfalfa cells (**Figure 6**) were consistent with the findings of Ulhassan et al. (2019). Previous research has discovered that Se can protect the chloroplast structure from degradation and promote photosynthesis (Alves et al., 2020). The present investigation revealed that the administration of Se treatment at a dosage of 100 mg/kg exhibited a significant preventive effect on the enlargement of thylakoid lamellae in chloroplasts. Furthermore, it effectively preserved the integrity of grana lamellae and capsule structure, restrained the augmentation of lipid spheres in terms of their number and volume, and delayed the disintegration of internal chloroplast structure. These findings are expected to facilitate the optimal functioning of chloroplasts in alfalfa. The aforementioned occurrence is also observed in other Se-fortified plants, such as tomatoes (Alves et al., 2020) and wheat (Semenova et al., 2017). Furthermore, a more vigorous intracellular protein processing process was detected when compared to the control, implying that under Se treatment, alfalfa cells maintained a favorable environment for nutrient accumulation *in vivo*. This ultrastructural evidence strongly demonstrates that Seon alfalfa has beneficial effects.

The small RNA sequencing data were subjected to bioinformatic analysis, which led to the identification of a significant number of mRNAs targeted by Se-induced miRNAs. These miRNAs were found to be enriched in KEGG pathways related to Carbohydrate metabolism and amino acid metabolism, including Photosynthesis (ko00195), Starch and sucrose metabolism (ko00500), Cysteine and methionine metabolism (ko00270), and Protein processing in the endoplasmic reticulum (ko04141). MiRNAs may influence biomass accumulation in alfalfa by regulating several photosynthesis-related genes. The transcriptional level of chlorophyll synthase (*GHLG*), a major enzyme involved in Chl recycling throughout the turnover of Chl-binding proteins of the PSI and PSII, was predicted to be inhibited by mtr-MIR2678-p5PsbY, which plays a crucial role in ensuring the stable assembly and photo-protection of the oxygen-evolving complex protein cytochrome b559 within the PSII core complex (von Sydow et al., 2016). PsbY expression may be regulated by lja-MIR171c-p3_2ss1AT17T to stabilize the PSII assembly and increase the photoreaction. Furthermore, it is plausible that miRNAs play a role in the process of plant carbon fixation within the Calvin cycle. The regulation of plant productivity through the control of Ribulose-1,5 bisphosphate carboxylase/oxygenase (*Rubisco*), a crucial enzyme and the initial stage in the Calvin cycle, was achieved by gma-mir172h-5p_ R-1 (Feng et al., 2020). Alfalfa’s carbon metabolism may also be controlled by miRNAs. Typically, the carbon dioxide that is assimilated via photosynthesis is initially stored as starch within the plant system. It was hypothesized that miRNAs played a role in the starch, sucrose, and TCA cycles, as evidenced by their targeting of genes associated with key enzymes such as starch synthase (*SS1*), beta-glucosidase (*BGLU*), sucrose synthase (*SUS*), trehalose-6-phosphate synthase (*TPS*), and aconitate hydratase (*ACO*). Given alfalfa’s ability to accumulate significant amounts of protein in comparison to other plants, it may be necessary to decrease carbohydrate biosynthesis and redirect more substrates towards protein synthesis (Annicchiarico et al., 2015). The GS/GOGAT pathway is the primary mechanism by which ammonium is absorbed into the carbon skeleton of plant cells to form glutamate (Glu) before its subsequent utilization (Renault et al., 2010). Nitrogen is digested in plants via the activity of Glutamine Synthetase (*GS*), which acts as a building block for all nitrogen-containing compounds in the plant (Seabra et al., 2010). *GS* was predicted to be the target gene of mdm-miR482b_R-1_2ss11CG20TG. The nitrogen present in alfalfa is believed to be derived from the uptake of nitrate ( ${\text{NO}}_{3}^{-}$ ) through the high-affinity nitrate transporter 2.1 (NRT2.1). The gene PC-5p-124278_36 is potentially involved in the regulation of NRT2.1 gene expression. According to reports, NRT2.1 is a significant constituent of the High-Affinity Transport System (*HATS*). Under most conditions, *NRT2.1* is the main contributor to the *HATS* (O’Brien et al., 2016). The present investigation suggests that gra-mir8782-p3_2ss13tc17ct has the potential to target glutamate decarboxylase (*GAD*). *GAD* catalyzes the formation of Glu γ- aminobutyric acid (*GABA*). *GABA* is essential for plant nitrogen homeostasis and stress resistance. *GABA* accumulation enhanced the total amino acid content of Arabidopsis (Renault et al., 2010). The present study suggests that miRNAs may exert a significant influence on Se response through modulation of gene expression levels associated with nitrogen, amino acid, and carbon metabolism, as evidenced by the aforementioned findings.

### 4.4 Involvement of miRNAs in Se biofortification of alfalfa by targeting DNA replication and repair processes

Previous research has mainly focused on examining the physiological impacts of Se on plants. Nevertheless, recent studies conducted on humans and animals have demonstrated that Se plays an essential role in the processes of DNA replication and repair. The inclusion of certain Se compounds in the diets of animals or humans in measured amounts has the potential to inhibit the formation of DNA adducts, as well as DNA or chromosomal breaks, and prevent chromosomal gain or loss (Ferguson et al., 2012). Furthermore, the administration of Se supplements may potentially reduce the occurrence of DNA adducts and chromosome breaks in studies of human malignancies, thereby mitigating the deleterious mutations that ultimately facilitate the development of cancer (Bera et al., 2013).

Recent research has indicated that the application of low concentrations of Na_2_SeO_3_ may enhance DNA replication and repair mechanisms, thereby stimulating maize growth (Dou et al., 2021). The present investigation aimed to examine the potential role of miRNAs induced by Se in regulating processes related to replication and repair. The KEGG analysis identified that a total of 39 and 69 mRNAs were enriched for downregulated and upregulated miRNAs, respectively, in the pathways of “Mismatch repair, DNA-templated (ko03430)”, “DNA replication (ko03030)”, “Nucleotide excision repair (ko03420)”, and “Homologous recombination (ko03440)”. The mini-chromosome maintenance protein (*MCM*) complex has been observed to function as DNA helicases during the process of DNA replication, thereby facilitating the opening up of the replication fork. This activity of the *MCM* complex serves as a licensing factor for DNA replication (Huang et al., 2019). *MCM6* was predicted to be the target gene of mtr-MIR2678-p3_1ss20CT in this investigation. *Dna*2 is a nuclease-helicase that is involved in Okazaki fragment processing, DNA end-resection during dsDNA break repair, and the resumption of DNA replication forks (*RFs*) that have become stalled (Appanah et al., 2020). In this study, it was hypothesized that mtr-MIR2678-p5 regulates the *Dna2* gene. Following the excision of the RNA primers, which is sealed by DNA ligase I, the Okazaki fragments generated during the synthesis of the lagging strand are joined by a DNA ligase, completing Okazaki fragment maturation (Becker et al., 2018). Apart from its involvement in the maturation of Okazaki fragments, *LIG1* also participates in the process of DNA repair (Chen et al., 2019). The fidelity of DNA replication is enhanced by the conserved DNA repair mechanism called mismatch repair (*MMR*), which corrects mispairs that occur during replication. This process involves genes such as *MLH1* and *MSH2* (Shell et al., 2007). *MLH1* was predicted to be the target gene of gra-MIR8782-p3_2ss13TC17CT. According to Recker’s findings, the regulator of telomere length helicase 1 (*RTEL1*) is essential for maintaining genomic integrity in plants (Recker et al., 2014). Furthermore, *RTEL1* was predicted to be the target gene of gra-MIR8782-p3_2ss13TC17CT. Based on the aforementioned results, it was hypothesized that the application of Se facilitated the growth of alfalfa by enhancing the processes of DNA replication and repair, thereby ensuring accurate DNA replication.

### 4.5 Vital roles of key miRNAs in the lncRNA/circRNA–miRNA–mRNA network

Despite the existence of several studies on the prevalence of lncRNAs/circRNAs in plants, there is a lack of research on the molecular mechanisms that regulate the role of lncRNAs/circRNAs in alfalfa Se biofortification. Specifically, the impact of lncRNA/circRNA-mediated modules on the enhancement of alfalfa growth and quality during Se biofortification remains unexplored. To investigate their involvement in the regulation of ncRNAs during the process of Se biofortification in alfalfa, a total of 150 modules consisting of ncRNAs/circRNAs–miRNAs–mRNAs were identified under Se treatment, as depicted in **Figure 12**. qRT-PCR was used to confirm the accuracy of RNA-seq (**Figure 13**). LncRNAs and circRNAs, as novel regulatory factors, have a negative effect on the expression of miRNAs by acting as miRNA sponges, which then indirectly influences the expression of the genes that miRNAs target (Salmena et al., 2011; Paschoal et al., 2018).

In plants, signal transduction involves the generation of ROS via the activation of NADPH oxidases [Respiratory Burst Oxidase Homolog F (*RbohF*)]. *RbohF* is required for shoot sodium homeostasis in *Arabidopsis* under salt stress (Jiang et al., 2013). The upregulation of lncRNA MSTRG.100041.2 resulted in the inhibition of gra-MIR8782-p3_2ss13TC17CT, consequently leading to the enhancement of *RbohF* expression. The observed phenomenon facilitated the development of resistance in alfalfa under the influence of Se treatment. The activation of enzymes involved in the AsA-GSH cycle and ROS-MG detoxification by Se-supplementation resulted in the reduction of ROS, MDA, and methylglyoxal (MG) levels in *Brassica napus* (Ulhassan et al., 2019). Glyoxalase II (hydroxyacylglutathione hydrolase 2, *GLX2*) is a crucial enzyme in the glyoxalase pathway, which is necessary for plant antioxidation. Similarly, the increased expression of ciRNA13 in alfalfa indirectly stimulated the expression of the downstream gene *GLX2*. The ciRNA13-mtr-MIR2678-p5 modules were found to induce activation of the glyoxalase pathway and enhance antioxidant activity in alfalfa. The antioxidant capacity of alfalfa was found to be improved under Se treatment, as evidenced by the increased GSH contents and GSH-PX activities, as illustrated in **Figure 3**. Proteostasis is a crucial element in ensuring optimal cellular performance, as it involves the precise regulation of various processes such as protein synthesis, folding, assembly, trafficking, and degradation. The protein denoted as Heat shock protein 70 (*Hsp70*) plays a pivotal role in the processes of protein folding, disintegration, and degradation. It was discovered that the lncRNA MSTRG.92275.3 could perform the function of a miRNA sponge by binding mtr-miR396a-5p. This enabled it to regulate the expression of *HSP70* at the same time. The *HSP40*, *HSP60*, and *HSP70* chaperone families were found to be among the most repressed, with *HSP70s* exhibiting the greatest degree of suppression overall (Fernández-Fernández et al., 2017). Thus, it can be concluded that the elevated expression of lncRNA MSTRG.92275.3 in alfalfa is associated with increased protein content via the MSTRG.92275.3-mtr-miR396a-5p-HSP70 modules. This factor has been shown to have a positive impact on the high quality of alfalfa (**Figure 4**).

The findings indicate that the modulation of signal transduction, glyoxalase pathway, and proteostasis by lncRNA/circRNA–miRNA–mRNA modules results in an improved antioxidant capacity and increased protein content in alfalfa. The activity of these modules explains why treatment with Se is beneficial to the growth of high-quality alfalfa.

## 5 Conclusion

**Figure 14:**
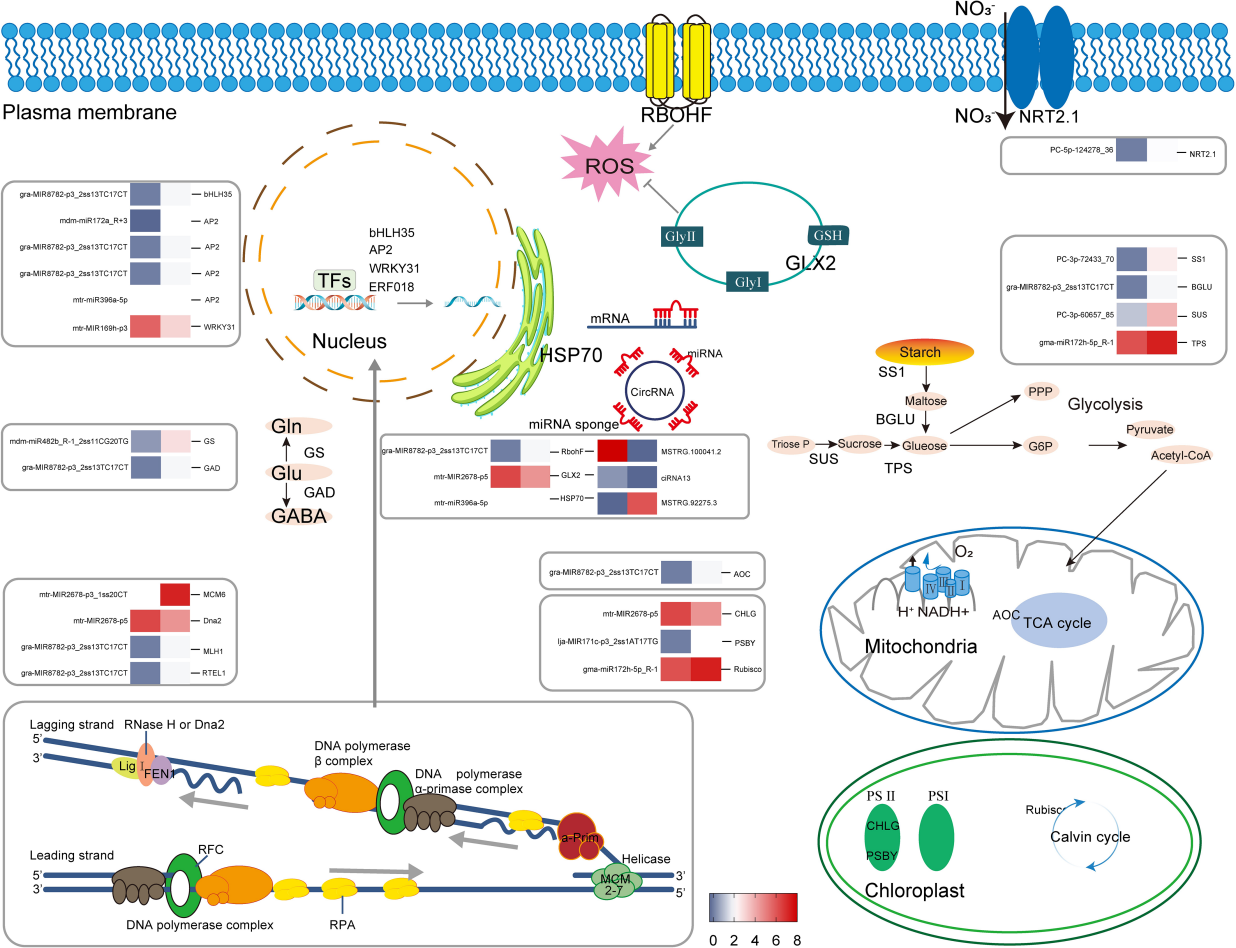
Precise regulation of gene expression by non-coding RNAs in alfalfa under Se fortification. Gene expression was log_2_ transformed.

A schematic diagram was drawn to illustrate the effects of Se (IV) on agronomy, quality, physiology, anatomy, and RNA-Seq (**Figure 14**). Se treatment effectively increased Se content, biomass accumulation, and protein levels in alfalfa. The increased antioxidant capacity aided in the maintenance of low ROS levels, hence promoting alfalfa stress resistance and intracellular environment stability. The increase in photosynthesis contributed to the biomass accumulation in alfalfa. DE-miRNA expression levels and their targets also contribute to the biomass accumulation and growth of high-quality alfalfa by influencing transcription factors, DNA replication and repair mechanisms, photosynthesis, carbohydrate metabolism, and protein processing. The findings of this investigation indicate that the activity of lncRNA/circRNA–miRNA–mRNA modules contributes to the enhancement of antioxidant capacity and protein content in alfalfa, which is achieved through the modulation of signal transduction, the glyoxalase pathway, and proteostasis. Collectively, these regulatory mechanisms facilitate the enhancement of hay yield, protein content, and RFV in alfalfa. The findings could be a useful resource for future research into the processes through which Se regulates plant development, biomass accumulation, and stress responses.

## Data availability statement

RNA-seq data are available at the NCBI SRA database (https://www.ncbi.nlm.nih.gov/geolquery/acc.cgi?acc=GSE192349) underaccession number GSE192349.

## Author contributions

QW and JH designed the experiments. TL, YL, and HH performed the qPCR verification. QW and JH carried out the experiment and analyzed the data. QW and JH wrote the manuscript. YS revised the manuscript. All authors contributed to the article and approved the submitted version.

## Supplementary material

Supplementary Table S1 List of primers used in qRT-PCR.

Supplementary Table S2 Identification of known and novel miRNA in alfalfa under Se biofortification.

Supplementary Table S3 List of 62 DEmiRNAs in alfalfa under Se biofortification.

Supplementary Table S4 List of 1414 DElncRNAs in alfalfa under Se biofortification.

Supplementary Table S5 List of 5 DEcircRNAs in alfalfa under Se biofortification.

Supplementary Table S6 Targeted mRNAs of these DEmiRNAs in alfalfa under Se biofortification.

Supplementary Table S7 GO terms of targets of DEmiRNAs in alfalfa under Se biofortification.

Supplementary Table S8 KEGG enrichment scatterplot of miRNAs target genes.

Supplementary Table S9 Predicted ceRNA regulated target genes in alfalfa under Se biofortification.
